# Weight change and risk of incident type 2 diabetes: short, medium and long-term follow-up in tehran lipid and glucose study

**DOI:** 10.1186/s12933-024-02297-w

**Published:** 2024-06-18

**Authors:** Samaneh Asgari, Davood Khalili, Fereidoun Azizi, Farzad Hadaegh

**Affiliations:** 1 Prevention of Metabolic Disorders Research Center, Research Institute for Endocrine Sciences, Shahid Beheshti University of Medical Sciences, P.O. Box 19395-4763, Tehran, Islamic Republic of Iran; 2 Endocrine Research Center, Research Institute for Endocrine Sciences, Shahid Beheshti University of Medical Sciences, Tehran, Iran

**Keywords:** Weight gain, Weight loss, Cohort study, Incident, Diabetes mellitus

## Abstract

**Background:**

Despite the high burden of obesity and Type 2 diabetes (T2DM) in the Middle East/West Asia region, the effect of weight change on the development of T2DM is poorly addressed. Therefore, we aimed to assess the impact of 3-year body weight change on incident of T2DM over 3-, 6-, and 9-year periods among Iranian adults.

**Methods:**

A total of 6930 participants (men = 2567) aged ≥ 20 years free of T2DM or cancer at baseline were included. Weight measurements were taken at baseline (2002–2005) and approximately 3 years later. Participants were categorized based on their weight change ratio into  ≥ 5% loss, stable (± 5%), and ≥ 5% gain. Generalized estimating equations (GEE), adjusted with age, sex, education levels, baseline measurements of fasting plasma glucose, weight, waist circumference, triglycerides to high-density lipoprotein cholesterol ratio, family history of diabetes, current smoker, hypertension, and prevalent cardiovascular disease were applied to estimate the Odds ratios (ORs) and 95% confidence intervals (CIs) of weight change categories for incident T2DM, considering stable weight as a reference.

**Results:**

During median follow-ups of 3-, 6-, and 9-year, 295, 505, and 748 cases of T2DM occurred, respectively. Weight gain of ≥ 5%, as compared to stable weight group (± 5%), was associated with increased T2DM risk, with ORs of 1.58 (95% CI 1.16–2.14), 1.76 (1.41–2.20), and 1.70 (1.40–2.05) for the 3-, 6-, and 9-year follow-ups, respectively, in multivariable analysis; corresponding values for weight loss ≥ 5% were 0.48 (0.29–0.80), 0.57 (0.40–0.81), and 0.51 (0.38–0.68), respectively. This association persisted even after adjusting for attained weight. Subgroup analysis showed consistent associations across age, gender, and body mass index categories.

**Conclusion:**

Weight gain and loss of ≥ 5% were associated with increased and decreased risks of incident T2DM, respectively, regardless of attained weight. This association was consistent over various follow-up durations among the Iranian population as recommended by guidelines.

**Supplementary Information:**

The online version contains supplementary material available at 10.1186/s12933-024-02297-w.

## Background

While diabetes has become a serious health concern throughout the world, the Middle East and North Africa (MENA) ranked as the first region with the highest prevalence of type 2 diabetes mellitus (T2DM) in 2021 globally (i.e., 16.2%). According to the 10th edition of The International Diabetes Federation (IDF) report in the MENA region, Iran stood out as the third-highest country in terms of both prevalence and the number of people with diabetes in 2021, with 5.5 million [1].

Several traditional risk factors are associated with incident T2DM [2], among which overweight/obesity shows a greater contribution to the development of T2DM in several studies, with approximately 60–90% of patients with T2DM being obese [3–5]. Notably, the American Diabetes Association (ADA) recommended that 3–7% weight loss is an effective strategy to effectively delay the progression of T2DM in prediabetes people [4]. In this line, Japanese individuals [6] who had a body mass index (BMI) gain of 5.0% or more had a 1.33-fold higher risk of diabetes compared to those with stable BMI values (− 5.0% to + 5.0%) during a median 2.5-year follow-up. In another study among the 8548 American population, weight gain over 11 kg during 9 years was significantly associated with increased risk of incident T2DM only in whites during 9-year follow-up [7]. The role of weight change and incident T2DM have been studied mostly in East Asian [6, 8–10], European, and American populations [7, 11, 12]. As the differences in body composition depend on the different ethnicities [13, 14], these studies are not directly transferable to the Middle East/West Asia region. Moreover, the short- and long-term effect of weight change on incident T2DM in one study design is less addressed in previous studies excluding post-trial follow-up of a few landmark interventional studies [15, 16].

Regarding the existence of a notable knowledge gap concerning the association between weight change and incident T2DM, we examined the impact of weight change on incident T2DM during 3-, 6-, and 9-year follow-ups among an Iranian population.

## Methods

### Study design and study population

**Fig. 1:**
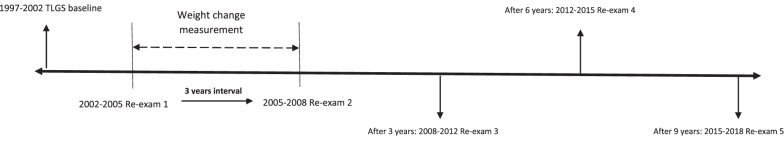
Timeline of the study design: tehran lipid and glucose study

The Tehran Lipids and Glucose Study (TLGS) is a community-based longitudinal cohort study conducted in District 13 of Tehran within a general urban population in Tehran. The study aims to determine the prevalence and incidence of non-communicable diseases and related risk factors among individuals aged ≥ 3 years. The initial registration phases occurred in 1999–2002 (TLGS baseline measurement) and cohort re-examinations were conducted at 3-year intervals, re-exam 1:2002–2005; re-exam 2:2005–2008; re-exam 3:2009–2012; re-exam 4:2012–2018; re-exam 5:2015–2018; and re-exam 6:2019–2022. The design and methodology of the TLGS study have been reported elsewhere [17]. As shown in Fig. 1, for the current study, measurements of re-exam 1 were set as the baseline, and re-exam 2 was considered as the second stage to calculate the weight change status of participants. Later, participants were followed up for 3-, 6-, and 9 years for the incident of T2DM.

Of the total of 8566 individuals ≥ 20 years who participated in 2002–2005, those who experienced T2DM at re-exams 1 and 2 were excluded (n = 1293). After removing pregnant women (n = 107), individuals with a history of cancer (n = 42), or hospitalized within the last 3 months before study measurements (n = 194), 6930 were eligible for this study. To address missing values (Table S1) in the present study, the mean of 10 imputed datasets through multiple imputation (MI) by chained equations (MICE) was considered to create a single imputation (SI) file for further estimation [18].

### Clinical and laboratory measurements

Demographic information, medication usage, cancer history, hospitalization history during the last 3 months before the study, family history of diabetes (FH-DM), prevalence of cardiovascular disease (CVD), smoking behaviors, and educational levels were obtained through validated questionnaires administered by interviewers during visits. Weight was recorded using a digital scale with participants wearing minimal clothing and no shoes, rounded to the nearest 100 g. Following a 15-min rest, systolic and diastolic blood pressure (SBP and DBP) measurements were taken as the mean of two readings on the right arm, using a standard sphygmomanometer. Morning blood samples were collected from 12-h fasting participants, and those not using glucose-lowering medications ingested 75 g of anhydrous glucose for a 2-h post-challenge plasma glucose (2h-PCG) assessment. Additional laboratory measurements, including fasting plasma glucose (FPG), 2h-PCG, triglycerides (TG), and high-density lipoprotein cholesterol (HDL-C), were previously detailed [19].

### Variable definitions

In the current study, T2DM was defined as having FPG ≥ 7 mmol/L and/or 2h-PCG ≥ 11.1 mmol/L or the use of anti-diabetic medications. TG to HDL-C ratio was calculated by dividing the values of TG by HDL-C. Moreover, hypertension was defined as SBP ≥ 140 mmHg or DBP ≥ 90 mmHg or the use of antihypertensive medications. Educational levels were considered into three groups: < 6 years, ≥ 6 and < 12 years, and ≥ 12 years of formal education. Current smoker was defined as who used any tobacco product (cigarette, pipe, and water pipe) at the time of examination. Prevalent CVD was defined as a positive history of acute coronary syndrome leading to CCU admission, history of percutaneous coronary intervention (PCI), coronary artery bypass graft (CABG), angiographic proven coronary artery disease, or history of stroke events.

Relative weight change (%) was calculated as:

$$\frac{{weight }_{re\_exam 2} -{weight }_{re\_exam 1} }{{weight }_{re\_exam 1}}\times 100$$

Furthermore, the weight change percentage is categorized as: (1) decreasing ≥ 5%; (2) ± 5% weight change (reference group: stable); (3) increasing ≥ 5% (4).

We decided to use weight rather than BMI due to the marginal changes in height over 3 years. BMI primarily reflects variations in weight, and using weight directly provides a more accurate change without the influence of height fluctuations.

### Statistical analysis

Baseline characteristics across weight change categories are shown as mean (standard deviation: SD) for normally distributed continuous variables, median (interquartile range: IQR) for skewed variables, and number (%) for categorical variables.

The data were reanalyzed using marginal models for longitudinal data, so the subjects were repeatedly considered over time in triennial periods for three re-exams. Generalized estimating equations (GEE) were carried out with a logit link function to estimate adjusted marginal means for the incident of T2DM over time by weight change categories. Three models were designed in the multivariable analysis. Model 1 included weight change groups, gender, and age; Model 2 was additionally adjusted for baseline FPG and initial weight; and Model 3 was further adjusted with baseline measurements of WC, TG/HDL-C, FH-DM, current smoker, hypertension, prevalent CVD, and education levels. In GEE models, further adjustments were made for time and interaction of time × weight change categories in all three models [20]. Additionally, the incidence level of T2DM in each re-exam was plotted by weight change categories. Moreover, all analyses repeated according to the reclassifications of weight change categories according to the Stevens et al. [21] recommendation with a minimum of 3% changes.

Statistical analysis was performed using Stata (version 17 SE) and P-values ≤ 0.05 were considered statistically significant.

## Results

**Fig. 2:**
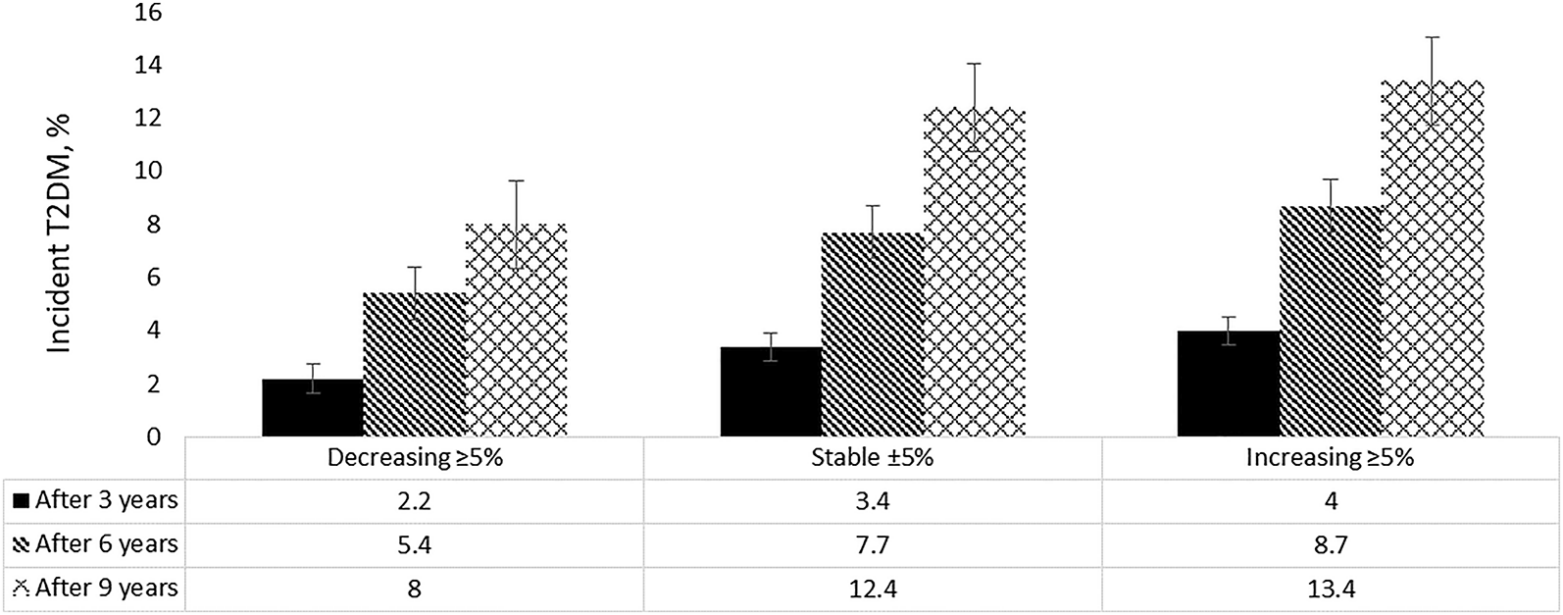
Incidence of type 2 diabetes according to the weight change groups at each follow-up: tehran lipid and glucose study 2002 − 2018

Characteristics of the participants across weight change categories during re-exam 1 and re-exam 2 are shown in Table 1. Approximately 70% of the participants maintained a consistent weight (± 5%) during the first three years of follow-up. Moreover, 9.1 and 21.9% of the total participants had more than 5% weight loss and weight gain, respectively. As predicted, weight loss ≥ 5% was associated with reduced SBP, DBP, FPG, and TG levels after 3 years, the level of metabolic risk factors was higher among individuals who had weight gain ≥ 5%. For those classified in the weight gain category, the median increase in weight was 5 kg, while individuals in the weight loss category experienced a median weight decrease of 5 kg as well. Out of a total of 6930 participants, 295, 505, and 748 individuals experienced incidents of T2DM during the third, fourth, and fifth re-examinations, respectively. Figure 2 shows the distinctions among the groups in terms of the occurrence of incident T2DM across successive re-examinations.

**Table 1:** Baseline and first re-examination characteristics of study participants across weight change categories: Tehran Lipid and Glucose Study

	Decreasing ≥ 5% (n = 627)		Stable ± 5% (n = 4799)		Increasing ≥ 5% (n = 1504)	
	Re-exam 1	Re-exam 2	Re-exam 1	Re-exam 2	Re-exam 1	Re-exam 2
Age, year	45.4 (14.7)	48.2 (14.8)	45.1 (14.2)	47.9 (14.2)	38.1 (13.0)	41.0 (13.0)
Gender, female	406 (64.7)	406 (64.7)	2627 (68.7)	2627 (68.7)	790 (52.5)	790 (52.5)
Education levels, year						
< 6	194 (31.0)	164 (27.2)	1498 (31.3)	1067 (24.1)	282 (18.8)	211 (14.6)
≥ 6 and < 12	328 (52.3)	335 (55.5)	194 (30.9)	164 (27.2)	924 (61.6)	941 (65.0)
≥ 12	105 (16.7)	105 (17.4)	282 (18.8)	211 (14.6)	295 (19.6)	295 (20.4)
Weight, kg	74.1 (14.4)	67.5 (13.2)	71.7 (12.4)	72.9 (12.8)	68.0 (13.4)	74.2 (14.5)
BMI, kg/m^2^	28.7 (5.1)	26.2 (4.6)	27.6 (4.4)	27.8 (4.5)	25.3 (4.6)	27.6 (4.9)
WC, cm	92.9 (11.9)	87.2 (11.9)	92.1 (11.4)	92.4 (11.7)	86.0 (12.1)	91.4 (12.4)
SBP, mmHg	117.4 (19.4)	111.5 (18.6)	116.6 (17.6)	116.0 (17.6)	110.9 (14.8)	112.6 (15.7)
DBP, mmHg	75.2 (10.8)	71.3 (9.8)	75.2 (10.4)	74.2 (9.7)	72.2 (9.5)	73.6 (9.0)
FPG, mmol/L	5.0 (0.5)	4.9 (0.5)	5.0 (0.51)	5.0 (0.52)	4.9 (0.5)	4.9 (0.5)
2h-PCG, mmol/L	6.0 (1.6)	5.6 (1.5)	6.0 (1.5)	5.8 (1.6)	5.5 (1.5)	5.6 (1.5)
TG, mmol/L	1.5 (1.1–2.2)	1.2 (0.9–1.8)	1.5 (1.1–2.2)	1.6 (1.1–2.1)	1.3 (0.9–1.9)	1.5 (1.1–2.1)
HDL-C, mmol/L	1.0 (0.27)	1.1 (0.25)	1.0 (0.26)	1.1 (0.27)	1.0 (0.27)	1.1 (0.25)
Current smoker, yes	114 (18.2)	89 (14.2)	856 (17.8)	560 (11.7)	337 (22.4)	219 (14.6)
Physical activity < 600 MET-min/week	249 (41.0)	158 (25.2)	1864 (38.8)	1112 (23.2)	617 (41.0)	391 (26.0)
FH-DM, yes	108 (17.2)	108 (17.2)	825 (17.2)	825 (17.2)	308 (20.5)	308 (20.5)
Prevalent CVD, yes	34 (5.4)	54 (8.6)	217 (4.5)	275 (5.7)	47 (3.1)	68 (4.2)
Hypertension medication, yes	42 (6.7)	20 (3.2)	322 (6.7)	168 (3.5)	58 (3.9)	36 (2.4)

Re-exam 1 (2002–2005), Re-exam 2 (2005–2008); Data are reported as mean (SD) for normally distributed variables, median (IQR) for skewed (e.g. TG), and frequency (%) for categorical variables

*SD* Standard deviations, *IQR* Interquartile range, *BMI* body mass index, *WC* waist circumference, *SBP* systolic blood pressure, *DBP* diastolic blood pressure, *FPG* fasting plasma glucose, *2h-PCG* 2-h post-challenge plasma glucose, *TG* triglycerides; *HDL-C* high-density lipoprotein cholesterol, *FH-DM* family history of diabetes, *CVD* cardiovascular disease, *MET* metabolic task equivalent

Table 2 illustrates the multivariable odds ratios (ORs) and 95% confidence intervals (CIs) elucidating the association between categories of weight change (minimum 5%) and the occurrence of T2DM events. In comparison to individuals with a stable weight change (± 5%), after controlling for the potential confounding factors (model 3), ≥ 5% in weight gain was associated with a higher risk of incident T2DM for the 3-year [ORs (95% CI) 1.58 (1.16–2.14)], 6-year [1.76 (1.41–2.20)], and 9-year follow-up [1.70 (1.40–2.05)]. Regarding decreasing ≥ 5% compared with participants with stable weight change, after adjustment for age, sex, education levels, baseline measurements of FPG, weight, TG/HDL-C, FH-DM, current smoking, hypertension, and prevalence CVD (model 3), the OR (95% CI) for incident T2DM was 0.48 (0.29–0.80) for 3-year follow-up, 0.57 (0.40–0.81) for 6-year follow-up, and 0.51 (0.38–0.68) for 9-year follow-up. Moreover, the significant covariates in model 3 were FH-DM, gender, and baseline measurements of age, TG/HDL-C, WC, FPG, and hypertension with all p-values < 0.005 (data not shown). Additionally, our data suggest that 1 SD of weight (about 4.4 kg) gained during 3 years, increases the risk of developing T2DM by at least 26%, even after considering attained weight.

**Table 2:** Weight change association (minimum 5%) with incident diabetes through follow-up time: Tehran Lipid and Glucose Study, 2002–2015

	After 3 years			After 6 years			After 9 years		
	E/N	OR (95% CI)	p-value	E/N	OR (95% CI)	p-value	E/N	OR (95% CI)	p-value
Model 1									
Stable ± 5%	223/4799	Reference		371/4799	Reference		558/4799	Reference	
Decreasing ≥ 5%	17/627	**0.56 (0.34–0.91)**	**0.02**	33/627	**0.67 (0.48–0.93)**	**0.02**	44/627	**0.61 (0.46–0.80)**	** < 0.001**
Increasing ≥ 5%	55/1504	1.05 (0.78–1.42)	0.74	101/1504	1.17 (0.94–1.46)	0.16	146/1504	1.13 (0.95–1.35)	0.17
Model 2									
Stable ± 5%	223/4799	Reference		371/4799	Reference		558/4799	Reference	
Decreasing ≥ 5%	17/627	**0.54 (0.33–0.89)**	**0.01**	33/627	**0.64 (0.46–0.90)**	**0.01**	44/627	**0.58 (0.43–0.77)**	** < 0.001**
Increasing ≥ 5%	55/1504	1.18 (0.87–1.60)	0.29	101/1504	**1.31 (1.06–1.63)**	**0.01**	146/1504	**1.28 (1.07–1.54)**	**0.008**
Model 3									
Stable ± 5%	223/4799	Reference		371/4799	Reference		558/4799	Reference	
Decreasing ≥ 5%	17/627	**0.48 (0.29–0.80)**	**0.005**	33/627	**0.57 (0.40–0.81)**	**0.002**	44/627	**0.51 (0.38–0.68)**	** < 0.001**
Increasing ≥ 5%	55/1504	**1.58 (1.16–2.14)**	**0.003**	101/1504	**1.76 (1.41–2.20)**	** < 0.001**	146/1504	**1.70 (1.40–2.05)**	** < 0.001**
1-SD increase in weight change*, kg	295/6930	**1.26 (1.18–1.35)**	** < 0.001**	505/6930	**1.30 (1.23–1.37)**	** < 0.001**	748/6930	**1.30 (1.24–1.36)**	** < 0.001**

Models are based on generalized estimating equations (GEE) for longitudinal data with a logit link function and bold values are significant at 95% confidence level

Model 1: weight change, age, sex

Model 2: model 1 + baseline fasting plasma glucose + initial weight

Model 3: model 2 + waist circumference + triglycerides/high density lipoprotein cholesterol ratio + family history diabetes + current smoker + hypertension + prevalence cardiovascular disease + education

1-SD of weight change is 4.4 kg

*E/N* event/number

*Adjusted with covariates in model 3

Table S2 illustrates the reclassifications of weight change categories with a minimum of 3% changes. Compared with a stable group (± 3%), no significant association was found between weight change 3–5%, either decreasing or increasing, with 3- and 9-year incident diabetes; however, we observed that weight change with increasing 3–5% was associated with a higher risk of 6-year incident T2DM [1.32 (1.03–1.70)]. Similarly to our main analysis, weight loss and weight gain ≥ 5% were significantly associated with a lower and higher risk of diabetes, respectively across three time points.

**Fig. 3:**
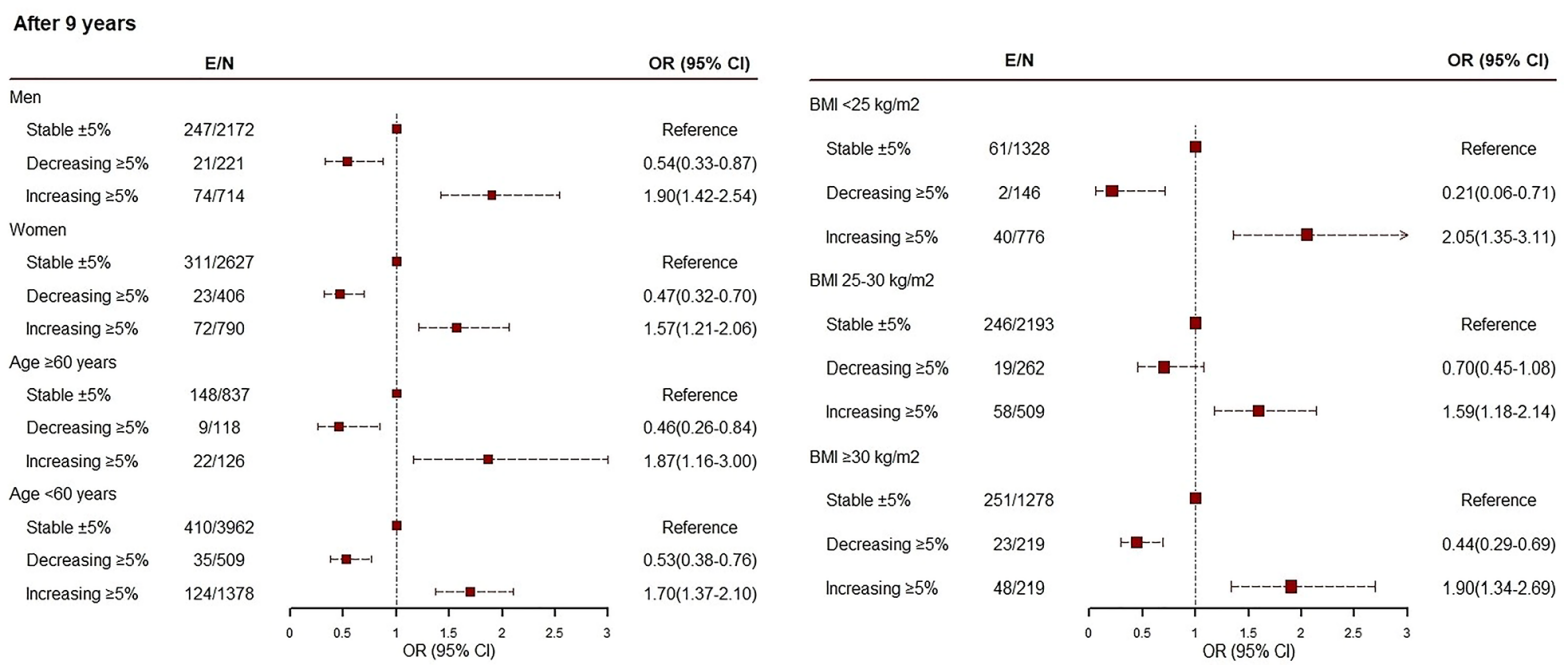
Weight change association with incident diabetes through follow-up by gender, age, and BMI categories over 9-year follow-up: tehran lipids and glucose study, 2002–2015. Models are based on generalized estimating equations (GEE) for longitudinal data with logit link function and adjusted for weight change categories, baseline age, sex (only for age-specific), fasting plasma glucose, weight, waist circumference, triglycerides/high-density lipoprotein cholesterol ratio, family history diabetes, current smoker, hypertension, prevalence cardiovascular disease, and education levels. *E* number of events, *N* number of participants. P-for interactions (Gender = 0.20; Age groups = 0.44; BMI categories = 0.98)

To show the robustness of our findings, a series of sensitivity analyses was done. First as presented in Table S3, we adjusted the attained weight instead of the initial weight to investigate the importance of weight change categories at a certain attained weight level. The results show that the association between categories of weight change and incident T2DM remained essentially similar; the OR (95% CI) of weight gain ≥ 5% in model 3 were 1.48 (1.09–2.03), 1.66 (1.32–2.08), and 1.60 (1.31–1.94) for incident 3-, 6-, and 9-year follow-up, respectively; corresponding values for weight loss ≥ 5% were 0.53 (0.32–0.89), 0.63 (0.44–0.90), and 0.56 (0.41–0.76), respectively. Second, as shown in Fig. 3, we examine the interaction between weight change categories (decreasing ≥ 5%; ± 5% weight change; and increasing ≥ 5%) and each of the age groups (≥ 60 years vs. < 60 years), gender (men vs. women), and baseline BMI category (< 25, 25–30, and ≥ 30 kg/m^2^) for the risk of 9-year incident T2DM. We found no significant interaction for weight change categories (p-for interaction age groups = 0.44, gender = 0.20, and BMI categories = 0.98), suggesting that the risk of incident T2DM as a function of weight change was similar among men and women, age below and over 60 years, and different BMI categories. Considering the limited number of events we did not consider 3-, and 6-year follow-ups.

## Discussion

In this large population-based study of Iranian urban residents, we found four key findings. First, weight gain ≥ 5% was associated with a minimum 60% higher risk of incident T2DM throughout the 3-, 6-, and 9-year follow-up periods even after adjustment for the participants’ initial weights and well-known diabetes risk factors. Second, weight loss ≥ 5% from the baseline was associated with a risk reduction of over 40% for the incident diabetes during the same follow-up periods. Third, the association between weight change (gain or loss) and incident T2DM did not change after adjustment for attained weight in place of initial weight. Fourth, subgroup analysis showed that the association between weight change and incident diabetes was robust in three sensitivity analyses including age, gender, and BMI categories.

Comparing our results with other studies in this field is not simple and poses some challenges. There were some differences between these studies regarding the duration of follow-up (ranging from 2.5 to 10 years), weight change duration (ranging from 1 to 20 years), weight measurement (self-report or measured), and weight change categorization (absolute change or relative change). Furthermore, variations exist in the analysis approach and level of adjustments for confounders.

Numerous research studies have shown that weight gain increases the risk of developing diabetes [6–11]. In our study, we found that 4.4 kg weight gain during 3 years increases the risk of developing diabetes by 26–30% during short and long-term follow-up. These results are in line with the findings reported by Jung et al. [9], demonstrating that each 2.8 kg weight gain over 2 years was associated with a 28% increased risk of incident diabetes during a 5-year follow-up period in the Korean population [9]. In another study, Xu et al. [22] found that each 10-kg weight gain over 30 years increases the risk of incident T2DM among the Chinese population by about 40%. With a similar weight gain among the Japanese population, the risk of incident T2DM was more than threefold over a 20-year follow-up period [10]. In another study among a cohort of US adults, Ford et al. [7] demonstrated that an increase in weight gain > 11 kg over 9 years was associated with an elevated risk of developing diabetes. Based on the change ratio, our results suggest that a weight gain < than 5% over three years does not yield significant short- or long-term implications for the incident T2DM in the general population. Conversely, a weight gain ≥ 5% is linked to at least a 60% rise in the risk of developing T2DM. This aligns with Ohno et al. [6] research, which showed that an increase of ≥ 5% in BMI over 2.5 years, compared to a stable change group, was significantly associated with a 33% higher risk of developing T2DM and reducing by at least 5% from the initial BMI was associated with an 18% reduction in the risk of incident T2DM.

Although several studies have demonstrated the association between weight loss and the incidence of diabetes [6, 9, 10], results have been inconsistent. In our data analysis weight loss ≥ 5% showed a suggestive 52%, 43%, and 49% decreased risk of incident diabetes during 3-, 6-, and 9- year follow-up, respectively. In line with ours, results from the Japanese population show that BMI loss ≥ 5% over 2.5 years, decreased the risk of 5-year incident diabetes by 18% [6]; however, reviewing cohorts conducted among American [7] and Dutch populations [11], did not find a significant association between weight loss and incident T2DM. Moreover, post-trial monitoring of the Diabetes Prevention Program Outcomes Study [15] and the China Da Qing Diabetes Prevention Study [16] found that the beneficial impact of lifestyle intervention for the prevention of diabetes persists for the period as long as ten and twenty years, respectively. Similarly, in our observational study, we found that a 3-year weight change reduced the risk of incident T2DM during a ten-year follow-up.

Research indicates that lifestyle or medical interventions are playing a higher role in weight loss which causes a lower rate of incident diabetes [4]. Results from the Look AHEAD trial show that in the intensive lifestyle intervention group, about 5% weight loss during 8 years improves glycemic control in people with diabetes [23, 24]. Besides, the effect of weight change in general population cohorts without interventions was also discussed [7–11, 25], and generally, no significant benefits of weight loss on risks of developing T2DM were reported. This risk reduction might be related to the improved nutrition quality of Iranian adults including an increase in total dietary Dietary Approach to Stop Hypertension (DASH) score, consumption of whole grains, legumes, nuts, and seeds, and a decrease in consumption of refined grain, and solid fats over a decade follow-up [26]. In the current study, the impact of physical activity on weight change was not examined, although the level of physical activity among Iranians has not improved over the past decade [27]. Importantly, we excluded individuals with cancer or recent hospitalizations within the last three months at the beginning of the study to address the potential impact of comorbidities on weight reduction.

To explore whether, at a certain level of attained weight, weight change history is important we adjusted attained weight, an issue that was poorly addressed in previous studies. We found that weight gain or loss has a significant impact on the incidence of T2DM, independent of attained weight measurements. In line with our findings, Kaneto et al. [10] demonstrated that weight changes over 5 years remained significantly associated with the occurrence of diabetes even after adjusting for final weight among 13,700 Japanese population aged 35–55 years. However, in the Dutch population-based Doetinchem cohort study, the significant association between weight change and the incidence of diabetes reached null [11].

In our subgroup analysis, we did not find an effect modification of gender, age, and BMI categories on incident diabetes. Similarly, the pooled cohort study conducted in Germany did not show the effect of modification of sex for the incidence of T2DM. Moreover, in the Japanese cohort, Ohno et al. [6] reported that both BMI gain and loss of ≥ 5% increased and decreased the risk of developing incident T2DM over 2.5 years to the same level, across normal weight, overweight, and obese individuals, respectively.

Our study strengths include the utilization of objectively measured weight data in follow-up re-examinations, distinguishing us from several studies that rely on self-reported data questionnaires, susceptible to recall information bias. Furthermore, our evaluation encompassed short, medium, and long-term follow-ups for incident diabetes. However, it is imperative to acknowledge certain limitations. Firstly, due to the absence of HbA1c data, diabetes was defined solely based on FPG and 2h-PCG. Secondly, the measures of weight change do not differentiate between alterations in lean or fat mass. Thirdly, since nutrition information was not assessed at re-exam 1 and only among a limited sample of participants in re-exam 2 (≈ 20%), we were not able to examine the impact of change in dietary behavior and calorie intake on observed weight change. Lastly, our study population is confined to residents of Tehran, a metropolitan city, sharing a uniform ethnicity; hence, caution should be exercised in generalizing our findings to rural populations or other ethnicities.

In conclusion, our data suggest that each 4.4 kg of weight gain during 3 years increases the risk of developing T2DM by at least 26%, even after considering attained weight. Moreover, the beneficial effect of weight loss is sustained for a decade follow-up. Care should be considered regarding an individual’s weight change, and lifestyle interventions are highly recommended to achieve ≥ 5% weight loss to prevent T2DM.

### Supplementary Information

Additional file1 (DOCX 27 kb)

## Data Availability

The datasets used and/or analyzed during the current study are available from the corresponding author upon reasonable request.

## Abbreviations

MENA: Middle East and North Africa
T2DM: Type 2 diabetes mellitus
IDF: International diabetes federation
ADA: American diabetes association
BMI: Body mass index
TLGS: Tehran Lipids and Glucose Study
MI: Multiple imputation
SI: Single imputation
FH-DM: Family history of diabetes
CVD: Cardiovascular disease
CVD: Cardiovascular disease
SBP and DBP: Systolic and diastolic blood pressure
2h-PCPG: 2-Hour post-challenge plasma glucose
FPG: Fasting plasma glucose
PCI: Percutaneous coronary intervention
CABG: Coronary artery bypass graft
IQR: Interquartile range
GEE: Generalized estimating equations
ORs: Odds ratios
CIs: Confidence intervals
TG: Triglycerides
HDL-C: High-density lipoprotein cholesterol
DASH: Dietary approach to stop hypertension
