# Supplementary material for: Weight change and risk of incident type 2 diabetes: short, medium and long-term follow-up in tehran lipid and glucose study
Source: Cardiovasc Diabetol. 2024 Jun 18;23:207. doi: 10.1186/s12933-024-02297-w (PMC11186083; doi:10.1186/s12933-024-02297-w)
Supplement: Supplementary file 1 — Additional file1 (DOCX 27 kb) [file 12933_2024_2297_MOESM1_ESM.docx]

| Table S1: Missing frequencies | |
| --- | --- |
| Variables | **Per 100 missing-per variable** |
| - Hypertension baseline | 0.16 |
| - Triglycerides baseline | 0.2 |
| - High density lipoprotein cholesterol baseline | 0.21 |
| - FPG baseline | 0.20 |
| - 2h-PCG baseline | 0.42 |
| - Waist circumference | 0.42 |
| - Current Smoking | 0.16 |
| - Education | 0.15 |
| - Weight baseline | 0.42 |
| - Weight re-exam 1 | 1.57 |
| - T2DM re-exam 2 | 1.59 |
| - T2DM re-exam 3 | 1.56 |
| - T2DM re-exam 4 | 1.82 |
| - T2DM re-exam 5 | 2.45 |
| FPG: fasting plasma glucose; 2h-PCG: 2-hour post-challenge plasma glucose; WC; waist circumference.   - Missing per covariates is calculated by the number of missing per covariate/(total population × total number of covariates) - Total number of used covariates =13; Total population in baseline: 8556 | |

| Table S2: Weight change association (minimum 3%) with incident diabetes through follow-up: Tehran Lipid and Glucose Study, 2002-2015 | | | | | | | | | |
| --- | --- | --- | --- | --- | --- | --- | --- | --- | --- |
|  | **After 3 years** | | | **After 6 years** | | | **After 9 years** | | |
|  | **E/N** | **OR (95% CI)** | **p-value** | **E/N** | **OR (95% CI)** | **p-value** | **E/N** | **OR (95% CI)** | **p-value** |
| Model 1 |  |  |  |  |  |  |  |  |  |
| - Stable ±3% | 165/3337 | Reference |  | 260/3337 | Reference |  | 399/3337 | Reference |  |
| - Decreasing 3-5% | 21/498 | 0.83(0.53-1.31) | 0.44 | 33/498 | 0.78(0.56-1.10) | 0.16 | 55/498 | 0.87(0.66-1.13) | 0.30 |
| - Decreasing ≥5% | 17/627 | **0.55(0.34-0.91)** | **0.02** | 33/627 | **0.67(0.48-0.93)** | **0.02** | 44/627 | **0.60(0.46-0.80)** | **<0.001** |
| - Increasing 3-5% | 37/964 | 0.94(0.66-1.34) | 0.75 | 78/964 | 1.14(0.89-1.45) | 0.29 | 104/964 | 1.06(0.87-1.30) | 0.57 |
| - Increasing ≥5% | 55/1504 | 1.05(0.78-1.42) | 0.74 | 101/1504 | 1.17(0.94-1.45) | 0.16 | 146/1504 | 1.13(0.95-1.35) | 0.17 |
|  |  |  |  |  |  |  |  |  |  |
| Model 2 |  |  |  |  |  |  |  |  |  |
| - Stable ±3% | 165/3337 | Reference |  | 260/3337 | Reference |  | 399/3337 | Reference |  |
| - Decreasing 3-5% | 21/498 | 0.79(0.50-1.26) | 0.32 | 33/498 | 0.75(0.53-1.07) | 0.11 | 55/498 | 0.84(0.63-1.12) | 0.23 |
| - Decreasing ≥5% | 17/627 | **0.52(0.31-0.86)** | **0.01** | 33/627 | **0.61(0.43-0.87)** | **0.006** | 44/627 | **0.55(0.41-0.74)** | **<0.001** |
| - Increasing 3-5% | 37/964 | 1.00(0.69-1.43) | 0.99 | 78/964 | 1.24(0.97-1.60) | 0.09 | 104/964 | 1.15(0.93-1.43) | 0.20 |
| - Increasing ≥5% | 55/1504 | **1.35(0.99-1.84)** | **0.057** | 101/1504 | **1.50(1.20-1.87)** | **<0.001** | 146/1504 | **1.43(1.19-1.73)** | **<0.001** |
|  |  |  |  |  |  |  |  |  |  |
| Model 3 |  |  |  |  |  |  |  |  |  |
| - Stable ±3% | 165/3337 | Reference |  | 260/3337 | Reference |  | 399/3337 | Reference |  |
| - Decreasing 3-5% | 21/498 | 0.76(0.47-1.21) | 0.24 | 33/498 | 0.72(0.51-1.03) | 0.07 | 55/498 | 0.80(0.60-1.07) | 0.14 |
| - Decreasing ≥5% | 17/627 | **0.48(0.29-0.80)** | **0.005** | 33/627 | **0.57(0.40-0.81)** | **0.002** | 44/627 | **0.51(0.38-0.68)** | **<0.001** |
| - Increasing 3-5% | 37/964 | 1.07(0.75-1.54) | 0.71 | 78/964 | **1.32(1.03-1.70)** | **0.03** | 104/964 | 1.22(0.98-1.51) | 0.08 |
| - Increasing ≥5% | 55/1504 | **1.57(1.16-2.14)** | **0.003** | 101/1504 | **1.76(1.41-2.20)** | **<0.001** | 146/1504 | **1.69(1.40-2.05)** | **<0.001** |
| Models are based on generalized estimating equations (GEE) for longitudinal data with logit link function.  Model 1: weight change, age, sex  Model 2: model 1+ baseline fasting plasma glucose+ initial weight  Model 3: model 2+ waist circumference+ triglycerides / high density lipoprotein cholesterol ratio+ family history diabetes+ current smoker + hypertension+ prevalence cardiovascular disease+ education  E/N: event/number; | | | | | | | | | |

| Table S3 : Weight change association with incident diabetes through follow-up after adjustment for attained weight: Tehran Lipid and Glucose Study, 2002-2015 | | | | | | | | |
| --- | --- | --- | --- | --- | --- | --- | --- | --- |
|  | **After 3 years** | | | **After 6 years** | | | **After 9 years** | |
|  | **OR (95% CI)** | **p-value** | **OR (95% CI)** | | **p-value** | **OR (95% CI)** | | **p-value** |
| - Stable ±5% | Reference |  | Reference | |  | Reference | |  |
| - Decreasing ≥5% | **0.53(0.32-0.89)** | **0.02** | **0.63(0.44-0.90)** | | **0.01** | **0.56(0.41-0.76)** | | **<0.001** |
| - Increasing ≥5% | **1.48(1.09-2.03)** | **0.01** | **1.66(1.32-2.08)** | | **<0.001** | **1.60(1.31-1.94)** | | **<0.001** |
| 1-SD increase in weight change*, kg | **1.21(1.12-1.31)** | **<0.001** | **1.30(1.22-1.38)** | | **<0.001** | **1.30(1.23-1.38)** | | **<0.001** |
| Models are based on generalized estimating equations (GEE) for longitudinal data with logit link function and adjusted for age+ sex+ attained weight + waist circumference+ triglycerides / high density lipoprotein cholesterol ratio+ family history diabetes+ current smoker + hypertension+ prevalent cardiovascular disease+ education  1-SD of weight change is 4.4 kg. | | | | | | | | |
